# The Adverse Effects and Nonmedical Use of Methylphenidate Before and After the Outbreak of COVID-19: Machine Learning Analysis

**DOI:** 10.2196/45146

**Published:** 2023-08-16

**Authors:** Hocheol Shin, Cindra Tri Yuniar, SuA Oh, Sujata Purja, Sera Park, Haeun Lee, Eunyoung Kim

**Affiliations:** 1 Evidence-Based Clinical Research Laboratory Department of Health Science and Clinical Pharmacy Chung-Ang University Seoul Republic of Korea; 2 Department of Pharmacology and Clinical Pharmacy School of Pharmacy Institut Teknologi Bandung Bandung Indonesia; 3 Regulatory Science Pharmacy College of Pharmacy Chung-Ang University Seoul Republic of Korea

**Keywords:** methylphenidate, attention-deficit/hyperactivity disorder (ADHD), social network services, adverse effect, nonmedical use, machine learning, deep learning, child, adolescent, psychiatric disorder

## Abstract

**Background:**

Methylphenidate is an effective first-line treatment for attention-deficit/hyperactivity disorder (ADHD). However, many adverse effects of methylphenidate have been recorded from randomized clinical trials and patient-reported outcomes, but it is difficult to determine abuse from them. In the context of COVID-19, it is important to determine how drug use evaluation, as well as misuse of drugs, have been affected by the pandemic. As people share their reasons for using medication, patient sentiments, and the effects of medicine on social networking services (SNSs), the application of machine learning and SNS data can be a method to overcome the limitations. Proper machine learning models could be evaluated to validate the effects of the COVID-19 pandemic on drug use.

**Objective:**

To analyze the effect of the COVID-19 pandemic on the use of methylphenidate, this study analyzed the adverse effects and nonmedical use of methylphenidate and evaluated the change in frequency of nonmedical use based on SNS data before and after the outbreak of COVID-19. Moreover, the performance of 4 machine learning models for classifying methylphenidate use based on SNS data was compared.

**Methods:**

In this cross-sectional study, SNS data on methylphenidate from Twitter, Facebook, and Instagram from January 2019 to December 2020 were collected. The frequency of adverse effects, nonmedical use, and drug use before and after the COVID-19 pandemic were compared and analyzed. Interrupted time series analysis about the frequency and trends of nonmedical use of methylphenidate was conducted for 24 months from January 2019 to December 2020. Using the labeled training data set and features, the following 4 machine learning models were built using the data, and their performance was evaluated using *F*-_1_ scores: naïve Bayes classifier, random forest, support vector machine, and long short-term memory.

**Results:**

This study collected 146,352 data points and detected that 4.3% (6340/146,352) were firsthand experience data. Psychiatric problems (521/1683, 31%) had the highest frequency among the adverse effects. The highest frequency of nonmedical use was for studies or work (741/2016, 36.8%). While the frequency of nonmedical use before and after the outbreak of COVID-19 has been similar (odds ratio [OR] 1.02 95% CI 0.91-1.15), its trend has changed significantly due to the pandemic (95% CI 2.36-22.20). Among the machine learning models, RF had the highest performance of 0.75.

**Conclusions:**

The trend of nonmedical use of methylphenidate has changed significantly due to the COVID-19 pandemic. Among the machine learning models using SNS data to analyze the adverse effects and nonmedical use of methylphenidate, the random forest model had the highest performance.

## Introduction

Attention-deficit/hyperactivity disorder (ADHD) is a widely diagnosed childhood psychiatric disorder [1,2]. Recently, ADHD has been increasingly diagnosed in adults as well as children and adolescents [3,4]. In the United States, about 10% of children aged between 4 and 17 years have been diagnosed with ADHD [5]. In 2007, the worldwide prevalence of ADHD was 5.29% [6]. Behavior therapy is the first-line treatment for ADHD in children 5 years of age or younger. Pharmacology therapy using methylphenidate is recommended if behavioral approaches do not give a significant outcome. From 6 years of age, methylphenidate is used as the first-line agent for ADHD [7]. Methylphenidate is a sympathomimetic drug that works by affecting the dopaminergic and noradrenergic systems. Noradrenaline and dopamine concentrations increase in the brainstem, midbrain, and frontal cortex. These effects are responsible for increased attention span and concentration [8]. However, through its pharmacology mechanism, methylphenidate can cause several adverse effects, including sleep problems, loss of appetite, and anxiety disorders. Furthermore, hallucinations and delusions may be caused by methylphenidate use [9,10]. A previous study showed that 1.2% of patients who used methylphenidate had serious adverse effects, including psychotic disorders. Moreover, 51.2% of patients experienced nonserious adverse effects, including sleep problems and loss of appetite. This study additionally noted that 7.3% of patients withdraw from methylphenidate use due to its adverse effects [9]. Methylphenidate has a potential risk of nonmedical use and is listed as a Schedule II substance in the United States [1,11,12]. It is likely to have effects resembling model illicit drugs, with a similar mechanism of action to cocaine and methamphetamine. The effect occurs because of the drug’s pharmacological action as a dopamine reuptake inhibitor and the subsequent synaptic dopamine increase observed after administration [11]. Therefore, methylphenidate has a potential risk of abuse. As it is a Schedule II substance, methylphenidate abuse can cause severe psychological or physical dependence. According to the National Survey on Drug Use and Health in the United States, 913,000 people aged >12 years abused methylphenidate in 2017. Moreover, 6.5% of the population aged between 18 and 25 years abused methylphenidate and amphetamine in 2018. The reason for methylphenidate abuse was to enhance focus while studying [12].

Drug use may have been affected by the spread of COVID-19. The outbreak of COVID-19 began in December 2019, and the World Health Organization soon declared it a pandemic. During the pandemic, people could not continue daily life, and industries were shut down. As a result, drug use was affected by these situations [13,14]. Hair analysis in Italy showed decreasing consumption of cannabis, benzodiazepine, and cocaine during and until the end of the lockdown period [15]. Self-reported surveys in the Netherlands also indicated declining trends of tobacco, alcohol, cocaine, and prescribed drug consumption [16]. Therefore, drug use evaluation as affected by the pandemic, as well as misuse of drugs, is important. However, it is difficult to determine abuse from randomized clinical trials and patient-reported outcomes. Moreover, most studies are conducted using surveys, but surveys have a response bias toward concealing abuse due to fear of detection [17]. As people share their purposes for using medication, patient sentiment, and the effects of medicine on social networking services (SNSs), the application of machine learning and SNS data can be a method to overcome the limitations [17-20]. A previous study analyzed the nonmedical use and adverse effects of methylphenidate on Twitter by applying the support vector machine (SVM) model. The SVM model had *F*-_1_ scores of 0.547 for nonmedical use and 0.733 for adverse effects [17]. However, the other SNS machine learning models could be applied to validate the effects of the COVID-19 pandemic on drug use. This study aims to determine the adverse effects and nonmedical use of methylphenidate and the change in frequency of nonmedical use depending on COVID-19 transmission. It also aims to evaluate the performance of several machine learning models for classifying methylphenidate use based on SNS data.

## Methods

### Step 1: Analysis of Adverse Effects and Nonmedical Use of Methylphenidate

#### Data Collection

**Figure 1:**
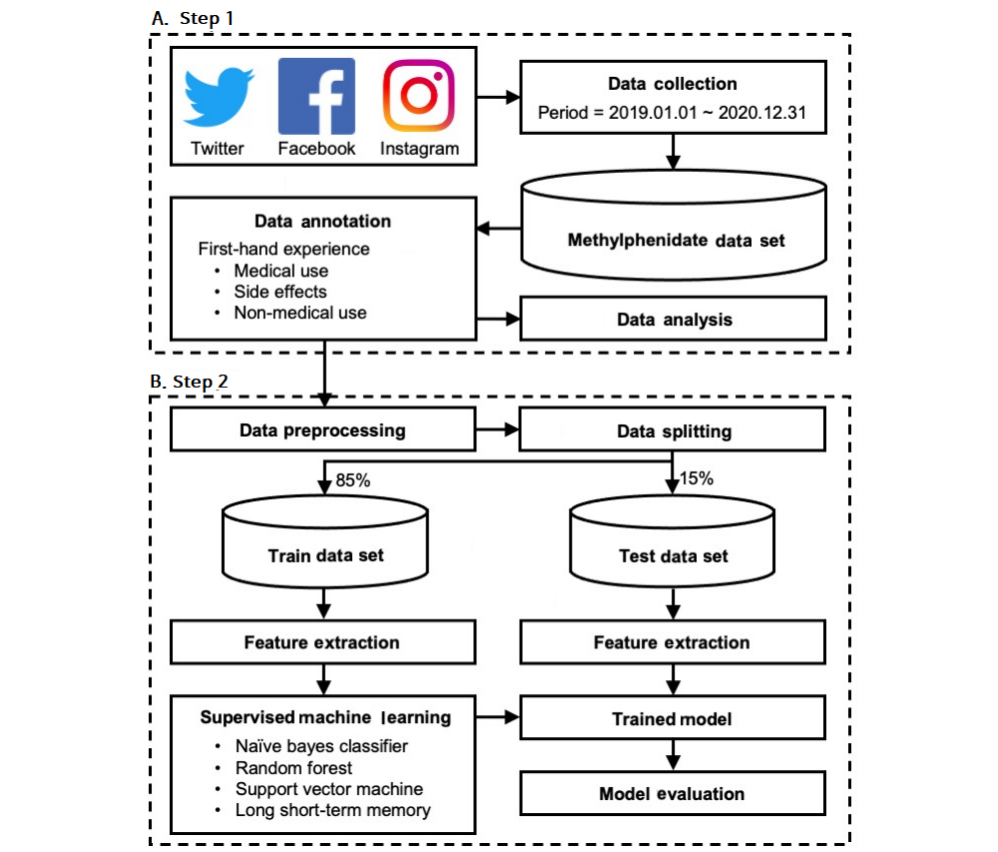
Schematic diagram of the study design.

This cross-sectional study collected and analyzed data on methylphenidate use from Twitter, Facebook, and Instagram between January 2019 and December 2020 (Figure 1A). These SNSs were used because they have many users and are representative of SNSs worldwide [21-23].

A web crawler was developed in Python software (version 3.7; Python Software Foundation) to collect the methylphenidate use data set because the Twitter application programming interface (API) policy was changed to require approval for crawling in 2021, and the SNS APIs have limitations on the collection period and information collected [24]. The following keywords were used to crawl for methylphenidate use: “Aptensio,” “Biphentin,” “Concerta,” “Daytrana,” “Equasym,” “Jornay,” “Medikinet,” “metadata,” “Methylin,” “QuilliChew,” “Quillivant,” “Ritalin,” “Rubifen,” and “Adhansia.” Data from all languages were collected and universally translated into English using the Google Translate program. Instagram and Twitter data mentioning the keywords were collected using the web crawler. However, as Facebook did not have sufficient keyword data, the Facebook data were manually collected.

#### Data Annotation

Collected data were annotated to analyze methylphenidate use by annotators manually. The collected data were identified as either (1) firsthand experience or (2) non–firsthand experience, and only firsthand experience data were used in the analysis. Firsthand experience was defined to mean that the writer of the SNS post had personally used methylphenidate [17,25]. Content annotated as non–firsthand experience were news, humor, research results, another person’s experience, other drugs, song lyrics, or other content based on the previous studies [17,25]. The data annotated as firsthand experience were additionally labeled as follows: (1) medical use, (2) adverse effects, and (3) nonmedical use. Medical use was defined as the use of a medication prescribed by a physician or pharmacist. All adverse effects were also collected. It was possible for medical/nonmedical use and adverse effects to appear in a single tweet. In these cases, the data could be classified as pertaining to both side effects and nonmedical use. In addition, drug abuse slang was considered as an abuse term for labeling because methylphenidate has the potential to produce effects similar to cocaine; thus, its abuse can be a gateway to illicit drugs [12,26]. The interannotator agreement was measured by Cohen Kappa, and disagreements were discussed with experienced clinical pharmacists [27,28].

#### Data Analysis Collected by Crawler From Twitter, Facebook, and Instagram

Initially, among the firsthand experience data, the frequency of side effects and nonmedical use of methylphenidate in the SNSs were analyzed monthly from January 2019 to December 2020. Interrupted time series analysis about nonmedical use of methylphenidate was conducted on “frequency” for a total of 24 months from January 2019 to December 2020. As a result of confirming the trend of “frequency” via a time plot, “frequency” showed a quadratic trend. To find out whether the trend of “frequency” changed after COVID-19, the period from January 2020 onward was designated as “corona outbreak,” and the trend of “frequency” before and after January 2020 was compared. For model identification using the Auto Regressive Integrated Moving Average model (ARIMA), the autocorrelation function (ACF) and the partial autocorrelation function (PACF) were obtained. As a result, the trend of “frequency” was determined by the autoregression (AR) model, written as AR(1). Therefore, since “frequency” is a data point with autocorrelation, regression analysis was performed using the generalized least squares method. Firsthand experience data were analyzed using Microsoft Excel (Microsoft Corp) spreadsheets, R software (R Foundation for Statistical Computing), and RStudio (version 4.1.2). Microsoft Excel was used to analyze the frequencies, percentages, and means of adverse effects and nonmedical use. The frequency of adverse effects and nonmedical use was analyzed by allowing duplication according to the subtopic. The *EpiTools* and *gmodels* packages in R and RStudio were applied to analyze the odds ratios (ORs) with a 95% CI, and a chi-square test was used to compare data before and after the COVID-19 pandemic. Coingestion in the subtopic of nonmedical use was the defined intake of methylphenidate with alcohol-related drinks or other stimulants, excluding drugs of abuse, such as cocaine and marijuana, as reported on the SNSs. Data regarding the use of cocaine and marijuana with methylphenidate or data with slang terms were identified as drug abuse. Recreational use was also defined as using methylphenidate to party or play games.

### Step 2: Performance Evaluation of the Machine Learning Models

#### Data Preprocessing

To improve the performance of the machine learning models, the collected SNS data were preprocessed (Figure 1B) [29-31]. Next, to perform the supervised learning, several features were generated, such as medical and nonmedical use terms, personal nouns, side effect terms, and so on. The selection of terms regarding adverse effects and nonmedical use were labeled according to the features reported by previous studies or were added if they appeared in the training data set [17,25]. For medical and nonmedical use terms, before generating the features of nonmedical use terms, natural language processing (NLP) was performed. Preprocessing was performed (1) to remove duplicate and null data, usernames, links, punctuation marks, numeric values, and stop words; (2) to convert terms to lowercase; and (3) for stemming. In detail, duplicate and null data, usernames, links, punctuation marks, and numeric values that did not contribute to classification and could hinder machine learning were removed [29]. Stop words, including “a,” “an,” “the,” and “with” were also removed. After removing these data, all words were converted to lowercase to reduce confusion and increase accuracy because machine learning recognizes lowercase and uppercase words as different. Finally, the Porter stemmer algorithm, which removes suffixes, was applied to reduce the text complexity. For example, “take” and “taking” contain the same information, but machine learning considers them different features [32-34]. Additionally, the long short-term memory (LSTM) model must unify the sentence length because of the model characteristics. As 99.05% (6280/6340) of the sentences had lengths ≤40 words, the sentence length in the LSTM model was unified to 40.

#### Machine Learning Models

This study compared the performance of 4 machine learning models using the *sklearn* and *TensorFlow* packages in Python: a naïve Bayes (NB) classifier, random forest (RF), SVM, and LSTM. The NB classifier, RF, and SVM are traditional models that are well-suited for classification [35]. LSTM was also applied because it effectively classifies and analyzes text data [36,37].

A set of hyperparameters was selected for each model for feature extraction techniques and structures to increase the performance and effectively control the complexity of the models [29]. The feature extraction techniques applied were bag-of-words (BoW) and term frequency-inverse document frequency (TF-IDF) [20,29]. Using a radial basis function kernel, SVM was trained to classify nonmedical use and side effects. To achieve a better performance, 2 hyperparameters of the SVM (ie, cost, gamma) were tuned. The hyperparameters were set for each model, and the remaining hyperparameters were set as default values. LSTM, a deep learning model, was structured in layers. The accuracy, precision, recall, and *F*-_1_ scores were calculated based on previous studies to evaluate the performance of the models [17,24]. True positives (TPs), false positives (FPs), true negatives (TNs), and false negatives (FNs) were calculated by comparing annotated results and predicted results according to the following equations (1)-(4):


Precision = TP/(TP + FP) **(1)**

Recall = TP/(TP + FN) **(2)**

Accuracy = (TP + TN)/(TP + TN + FP + FN) **(3)**

*F*-_1_ = (2× Precision × Recall)/(Precision + Recall) **(4)**

Due to the imbalance of these categorical data, the performance of the classifiers was evaluated using the *F*-_1_ score (ie, harmonic mean of precision and recall) instead of accuracy. The data set was randomly divided into 85% of the data set to train the models and 15% to evaluate their performance. In addition, the training data set had a very large number of negative samples. To compensate for this issue, 10-fold cross-validation was performed on the training data, and inverse weights were assigned to positive and negative samples [17,25].

### Ethical Considerations

Twitter, Facebook, and Instagram are major social networks. Under strict policies, they try to regulate the ethical use of their data and maintain the privacy of their users. The information needed for the processing steps has been collected and saved following their guidelines. All sensitive data were discarded after analysis.

## Results

### Overview

From January 2019 to December 2020, a total of 146,352 data points were collected from Twitter, Facebook, and Instagram (Table 1). Data annotated as firsthand experience comprised 4.3% (6340/146,352) of the data set, including medical use (2641/6340, 41.7%), adverse effects (1683/6340, 26.5%), and nonmedical use (2016/6340, 31.8%). The Cohen Kappa was 0.92, which means that the agreement was substantial. The word cloud indicates that people post about using methylphenidate for ADHD treatment or study and share their experiences of adverse effects, such as anxiety, depression, and loss of appetite (Figure S1 of Multimedia Appendix 1).

**Table 1:** Data set collected by a web crawler from Twitter, Facebook, and Instagram.

Data			Twitter (N=145,520), n (%)	Instagram (N=672), n (%)		Facebook (N=160), n (%)		Total SNSs^a^ (N=146,352), n (%)
Non–firsthand experience			139,255 (95.7)	599 (89.1)		158 (98.8)		140,012 (95.7)
**Firsthand experience**			6265 (4.3)	73 (10.9)		2 (1.2)		6340 (4.3)
	Medical use	2590 (41.3)		50 (68.5)	1 (50)		2641 (41.7)	
	Side effects	1660 (26.5)		22 (30.1)	1 (50)		1683 (26.5)	
	Nonmedical use	2015 (32.2)		1 (1.4)	0 (0)		2016 (31.8)	

^a^SNS: social networking service.

### Analysis of the Adverse Effects and Nonmedical Use of Methylphenidate

#### Frequency of Adverse Effects and Nonmedical Use

The data on adverse effects and nonmedical use were analyzed by allowing duplication (Table 2).

Psychiatric problems, including anxiety, depression, jitters, nervousness, panic, restlessness, tension, and worry, were the most frequent adverse effects of methylphenidate (521/1683, 31%). Sleep problems and loss of appetite accounted for a significant proportion of the adverse effects of methylphenidate at 18.7% (314/1683) and 14.8% (250/1683), respectively. Gastrointestinal and neurological problems were also frequent adverse effects at 7.7% (129/1683) and 5.4% (91/1683), respectively. In particular, a few hallucinations and delusions were discovered in the SNSs.

The frequency of nonmedical use of methylphenidate was also analyzed (Table 2). Study or work was the most frequent reason for nonmedical use (741/2016, 36.8%). Coingestion and drug abuse were additional nonmedical uses at 19.9% (401/2016) and 13% (262/2016), respectively. Methylphenidate was also occasionally abused for recreational purposes (3.5%, 70/2016). The data on recreational use indicated that the users took methylphenidate to party or play games for longer amounts of time.

**Table 2:** Data frequency of side effects and nonmedical use of methylphenidate in the SNSs^a^.

Topics and subtopics		Data frequency^b^, n (%)
**Side effects (N=1683)**		
	Psychiatric problems	521 (31)
	Sleep problems	314 (18.7)
	Loss of appetite	250 (14.8)
	Heart problems	138 (8.2)
	Gastrointestinal problems	129 (7.7)
	Neurological problems	91 (5.4)
	Sweating	21 (1.2)
	Eye problems	12 (0.7)
	Other	402 (23.9)
**Nonmedical use (N=2016)**		
	Study or work	741 (36.8)
	Coingestion	401 (19.9)
	Drug abuse	262 (13)
	Overdose	186 (9.2)
	Seeking or obtaining	118 (5.8)
	Recreational use	70 (3.5)
	Loss of weight	44 (2.2)
	Other	265 (13.1)

^a^SNS: social networking service.

^b^Data frequency of side effects and nonmedical use of methylphenidate was analyzed by allowing duplication.

#### Comparison of Nonmedical Use of Methylphenidate Before and After the COVID-19 Pandemic

The frequency of nonmedical use of methylphenidate was compared before and after the COVID-19 pandemic, as drug use has been affected by the COVID-19 pandemic. The OR for nonmedical use of methylphenidate after the pandemic was 1.02 (95% CI 0.91-1.15) compared to before the pandemic, indicating insignificant results. This indicates no difference in the frequency of nonmedical use before and after the pandemic. However, coingestion and drug abuse in nonmedical use have significantly changed (Table S1 of Multimedia Appendix 1). While the incidence of coingestion increased from 17.7% to 22.9% (**P*<.01*), that of drug abuse decreased from 16.5% to 8.3% (**P*<.001*) after the pandemic.

**Figure 2:**
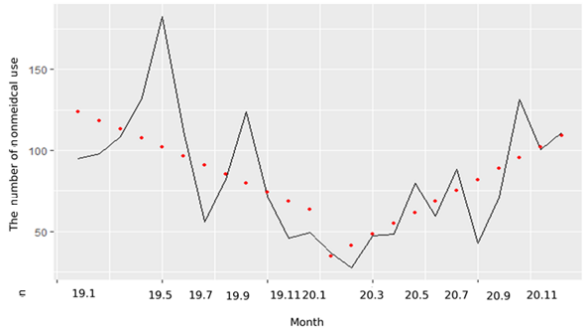
Distribution of data about nonmedical use of methylphenidate by month.

The distribution of nonmedical use of methylphenidate differed before and after the COVID-19 pandemic (Figure 2). The frequency of nonmedical use before the pandemic was the highest in May 2019 (182/2016, 9%). However, the frequency of nonmedical use reached its lowest point in February 2020 (29/2016, 1.4%), which was immediately after the COVID-19 outbreak. It increased in May 2020 (80/2016, 4%) and showed an increasing trend until October 2020. Interrupted time series analysis was conducted on “frequency” for a total of 24 months from January 2019 to December 2020. When evaluated by time plot, “frequency” showed a quadratic trend. To check whether the trend of “frequency” changed after COVID-19, the trend of “frequency” before and after January 2020 was compared by setting the point of January 2020 as “corona outbreak.” As a result of obtaining the ACF and PACF to identify the model using the ARIMA model, the trend of “frequency” was determined by the AR(1) model. Therefore, since “frequency” is a data point with autocorrelation, regression analysis was performed using the generalized least squares method. As a result of the analysis, before the outbreak of COVID-19 (2019), the frequency of nonmedical use of methylphenidate tended to decrease by 5.52 per month, but it was not significant (95% CI −12.08 to −1.04). However, after the outbreak of COVID-19 (2020), it was significant with a monthly increase of 12.279 (95% CI 2.36-22.20). Therefore, the trend of the frequency of methylphenidate nonmedical use has changed significantly due to the COVID-19 pandemic.

Table 3 shows the hyperparameters of the machine learning models.

The RF model with BoW and TF-IDF and the SVM model with TF-IDF produced the highest *F*-_1_ score of 0.75 (Table 4). In contrast, LSTM had the lowest *F*-_1_ score of 0.69. Because the adverse effects and nonmedical use of methylphenidate vary and these text patterns were randomly established, LSTM performed worse than the other models. The low recall for adverse effects was also responsible for their low *F*-_1_ scores. It is suggested that the models were less effective at filtering for false negatives. The performance of the RF model and the NB classifier was the same with BoW and TF-IDF, and the NB classifier and SVM performed slightly better with TF-IDF than BoW.

**Table 3:** Hyperparameters of the machine learning models.

Models and analysis type		Hyperparameters
**With BoW^a^**		
	NB^b^	N/A^c^
	RF^d^	n_estimates = 500
	SVM^e^	C = 10, gamma = 0.1
**With TF-IDF^f^**		
	NB	TF-IDF = character N-gram (range = (3, 4)), alpha = 0.1
	RF	TF-IDF = count, n_estimates = 500, max_depth = 300, random_state = 3
	SVM	TF-IDF = count
**Deep learning**		
	LSTM^g^	embedding layer = dimensions (40, 100), neurons of LSTM layer = 128, activation function of LSTM layer = “sigmoid,” neurons of dense layer = 3, activation function of dense layer = “softmax,” loss function = “sparse categorical crossentropy,” optimizer = “adam”

^a^BoW: bag-of-words.

^b^NB: Naïve Bayes.

^c^N/A: not applicable.

^d^RF: random forest.

^e^SVM: support vector machine.

^f^TF-IDF: term frequency-inverse document frequency.

^g^LSTM: long short-term memory.

**Table 4:** Performance of the machine learning models.

Model and class				Accuracy		Precision		Recall		*F*-_1_ score
**With** **BoW^a^**										
	**NB^b^**			0.71						
		0^c^			0.69		0.82		0.75	
		1^d^			0.65		0.60		0.63	
		2^e^			0.80		0.66		0.72	
		Macroaverage			0.72		0.69		0.70	
		Weighted average			0.72		0.71		0.71	
	**RF^f^**			0.76						
		0			0.75		0.89		0.81	
		1			0.75		0.54		0.63	
		2			0.79		0.78		0.79	
		Macroaverage			0.76		0.74		0.74	
		Weighted average			0.76		0.76		0.75	
	**SVM^g^**			0.72						
		0			0.66		0.93		0.77	
		1			0.77		0.44		0.56	
		2			0.82		0.68		0.74	
		Macroaverage			0.75		0.68		0.69	
		Weighted average			0.74		0.72		0.71	
**With TF-IDF^h^**										
	**NB**			0.73						
		0			0.70		0.84		0.76	
		1			0.72		0.60		0.65	
		2			0.80		0.68		0.73	
		Macroaverage			0.74		0.71		0.72	
		Weighted average			0.73		0.73		0.72	
	**RF**			0.76						
		0			0.75		0.89		0.81	
		1			0.75		0.51		0.61	
		2			0.79		0.79		0.79	
		Macroaverage			0.76		0.73		0.74	
		Weighted average			0.76		0.76		0.75	
	**SVM**			0.76						
		0			0.76		0.85		0.80	
		1			0.75		0.57		0.65	
		2			0.76		0.80		0.78	
		Macroaverage			0.76		0.74		0.74	
		Weighted average			0.76		0.76		0.75	
**Deep learning**										
	**LSTM^i^**			0.69						
		0			0.75		0.72		0.74	
		1			0.62		0.58		0.60	
		2			0.68		0.75		0.71	
		Macroaverage			0.68		0.68		0.68	
		Weighted average			0.69		0.69		0.69	

^a^BoW: bag-of-words.

^b^NB: Naïve Bayes.

^c^Class 0 refers to medical use.

^d^Class 1 refers to side effects.

^e^Class 2 refers to nonmedical use.

^f^RF: random forest.

^g^SVM: support vector machine.

^h^TF-IDF: term frequency-inverse document frequency.

^i^LSTM: long short-term memory.

## Discussion

### Principal Findings

This study analyzed the adverse effects and nonmedical use of methylphenidate and evaluated the change in frequency of nonmedical use before and after COVID-19 using data from Twitter, Facebook, and Instagram. It also evaluated the performance of machine learning to effectively classify methylphenidate use from SNS data by comparing the results of the machine learning models.

Psychiatric problems were the most frequent adverse effects. The clinical concern is that treatment with methylphenidate may increase the risk of psychosis. The incidence rate of psychosis was 1.78 episodes per 1000 person-years of drug exposure in the methylphenidate group in a cohort study using 2 databases in the United States [38]. Psychosis might camouflage ADHD symptoms, such as anxiety and mood disorders because the same brain region and neurotransmitter system are involved in ADHD [39]. Moreover, 2 studies demonstrated that psychiatric problems are still considered clinical concerns during methylphenidate consumption [38,40].

Sleep problems were found to be common adverse effects of methylphenidate in this study. Because they impact daytime attention and mood disorders, sleep problems are important adverse effects and must be managed continuously [41]. A review of the safety and tolerability of ADHD medications noted that insomnia was one of the most commonly reported adverse events associated with psychostimulant treatment [39].

Furthermore, gastrointestinal and neurological problems were also common adverse effects. While most adverse effects occur in similar proportions as in the methylphenidate clinical trial results in the United States, the proportion of gastrointestinal and neurological problems was lower than that in clinical trials [42]. A previous survey also indicated that the adverse effects of methylphenidate were different from clinical trials [43]. This may indicate that results from self-reporting, such as surveys, differ from clinical trial results. However, the difference between adverse effects data from clinical trials and surveys is acceptable because drugs can cause different responses within a large population. A pharmacovigilance system was applied to detect rare adverse effects, especially in postmarketing surveillance studies and spontaneous adverse drug reaction reporting [44].

The results of this study showed that nonmedical use for study or work had the highest frequency, followed by coingestion and drug abuse. Methylphenidate is widely used for academic achievement without a prescription by a physician [45-47]. The increasing motivation in patients with methylphenidate consumption was caused by increasing dopamine transporters and D2/D3 receptor availability [48]. In particular, the illicit use of methylphenidate to increase concentration is strongly associated with drug abuse (eg, cocaine abuse) [49]. Abusing, seeking, or obtaining methylphenidate, behaviors that are difficult to analyze via surveys, were significantly detected in the SNS data. This indicates that people use methylphenidate as a gateway for abuse and obtain the drug from peers or through doctor shopping [45,47]. Moreover, this study showed that some people use methylphenidate for recreational purposes. In a previous survey-based study, 107 of 164 students said they used methylphenidate to party for a longer time, which suggested that education should be provided to students who abuse methylphenidate [50,51].

Nonmedical use of methylphenidate was compared before and after the COVID-19 pandemic. The frequency of nonmedical use was not shown to be different before and after the pandemic. However, coingestion in nonmedical use was significantly higher after the pandemic because coingestion can be easier to abuse than other nonmedical uses. While drug abuse during the pandemic also generally increased because people stayed at home, as required due to social distancing protocols, stimulant abuse was reported to decrease after the COVID-19 pandemic in previous studies [13,52,53]. Similarly, this study indicated that the abuse of methylphenidate decreased after the COVID-19 pandemic. Drug abuse decreased because it became difficult to import the drug substance for the stimulant because of the disruption of raw material production [13]. The pandemic may also have contributed to the decrease in drug abuse because people could not get together for parties. A study in the Netherlands showed that the consumption of ecstasy, amphetamines, cocaine, nitrous oxide, ketamine, LSD, psychedelic mushrooms or truffles, GHB, 2C-B, 3-MMC, and 4-MMC (other than prescription drugs) decreased during the pandemic because of fewer social occasions (eg, going out, appointments, visits, parties, and other activities) [16].

The frequency of nonmedical use decreased from May 2019 to May 2020, which is the traditional examination period, and immediately after the COVID-19 outbreak [5,19]. The main reason for the decline in the nonmedical use of methylphenidate was the closure of schools and universities ordered due to COVID-19, which made typical academic classes impossible [54,55]. School closures may have directly affected the amount of nonmedical use. Moreover, the COVID-19 pandemic contributed to a 75% decrease in outpatient visits [14]. Patients had difficulty purchasing medicines because of the pandemic and social distancing. However, after the outbreak of COVID-19 in 2020, it was significant, with a monthly increase of 12.279 (95% CI 2.36-22.20). Therefore, although the frequency of methylphenidate nonmedical use has not changed significantly due to the COVID-19 pandemic, its trend has changed significantly.

This study had better *F*-_1_ scores than that of Kim and colleagues [17], in which the *F*-_1_ score for nonmedical use of methylphenidate was 0.547, and that for adverse effects of methylphenidate was 0.733. However, the precision scores were not better in this study than in the study by Kim and colleagues, which were 0.920 for adverse effects and 0.926 for nonmedical use. This suggests that this study was not more effective at filtering false positives.

The *F*-_1_ scores of the deep learning models in the studies by Li et al [24] and Hu et al [18] were higher than in this study at 0.93 and 0.86, respectively. This is due to similar text patterns and words in the data. As previous studies had specific subjects showing the detection of illicit drug dealers and stimulant abuse, their performance was higher than in this study. However, in this study, as in previous studies, RF outperformed the other models because the class planes that were linearly separated could not be used for the text data [18,24,29].

### Limitations

Most data collected from SNSs were obtained from Twitter (145,520/146,352, 99.4%), and Facebook and Instagram data accounted for only 0.6% (832/146,352). Thus, the results were biased only toward Twitter. These data proportions were caused by the policies of each SNS. Facebook and Instagram changed their policies to block searches for specific medicines and suspended offending accounts in April 2018 [24]. Searches and text may have been limited on Facebook and Instagram, and the search results may also have been hidden or deleted by the SNS policies. Moreover, about 9% of the teenagers in previous studies who potentially took methylphenidate use Facebook in the United States, and Instagram is not based primarily on text [21-23]. These factors may affect the volume of the collected data. Therefore, it is necessary to study data from other SNSs that are unmanaged and frequently used by students. Nevertheless, NLP techniques continue to develop rapidly, and new methods emerge one after another. However, the NLP method applied in this study was the method used in previous studies to reconfirm whether there was a similar pattern during a similar period compared to previous studies and to confirm the impact of COVID-19 [17]. In the future, results from new NLP methods are expected to generate advanced outcomes. Moreover, because we performed traditional supervised learning, each instance is associated with only a single class label. Although binary classification based on text structure is a relatively simple type of text classification, real-world SNS instances might have multiple semantic meanings simultaneously. If such multilabel learning is applied in future studies, we expect that it will reflect the real environment well for each instance [56,57].

Another limitation is that this study could not analyze changes in the nonmedical use of methylphenidate in 2021 when social restrictions and public alarm around the COVID-19 pandemic decreased, and it became a part of daily life. The distribution of nonmedical use in this study clearly indicates a change before and after COVID-19 transmission. However, as the analysis period was limited between January 2019 and December 2020, more recent nonmedical use could not be analyzed. Therefore, additional studies are needed to analyze how the status of nonmedical use has changed since 2021.

The results of this study are not generalizable and do not necessarily apply to the actual adverse effects and nonmedical use of methylphenidate. Most studies that rely on SNS data face this limitation [28]. The reason is that Twitter, Facebook, and Instagram restrict users aged <13 years, and this population is adept at using SNSs. Moreover, it cannot verify if users actually use the medications and experience adverse effects or if they use them for nonmedical purposes. However, despite this limitation, studies using SNSs can easily and effectively analyze the status of stimulant use.

### Conclusions

This study analyzed the adverse effects and nonmedical use of methylphenidate and evaluated the frequency and changes in the trend of nonmedical use frequency before and after the COVID-19 pandemic on Twitter, Facebook, and Instagram. This study also evaluated the performance of machine learning to classify methylphenidate use from SNS data by comparing the results of 4 different machine learning models. The results of this study will contribute to determining the status of methylphenidate use, and machine learning using SNS data will be useful in applications for automatic monitoring tasks. Because stimulants are potentially abused or used as a gateway to drug abuse, their use must be continuously managed. Therefore, future studies should seek to apply machine learning to other stimulants and other SNSs that are unmanaged and frequently used by students.

## Appendix

Multimedia Appendix 1 Supplemental table and figure.

## Abbreviations

ACF: autocorrelation function
ADHD: attention-deficit/hyperactivity disorder
API: application programming interface
AR: autoregression
ARIMA: Auto Regressive Integrated Moving Average
BoW: bag-of-words
FN: false negative
FP: false positive
LSTM: long short-term memory
NLP: natural language processing
OR: odds ratio
PACF: partial autocorrelation function
SNS: social networking service
SVM: support vector machine
TF-IDF: term frequency-inverse document frequency
TN: true negative
TP: true positive

## Data Availability

The data sets generated during and/or analyzed during this study are available from the corresponding author upon reasonable request.
