# Supplementary material for: The Adverse Effects and Nonmedical Use of Methylphenidate Before and After the Outbreak of COVID-19: Machine Learning Analysis
Source: J Med Internet Res. 2023 Aug 16;25:e45146. doi: 10.2196/45146 (PMC10468706; doi:10.2196/45146)
Supplement: Multimedia Appendix 1 [file jmir_v25i1e45146_app1.docx]

**Multimedia Appendix 1**

**Table S1. Comparison for nonmedical use of methylphenidate before and after the COVID-19 pandemic**


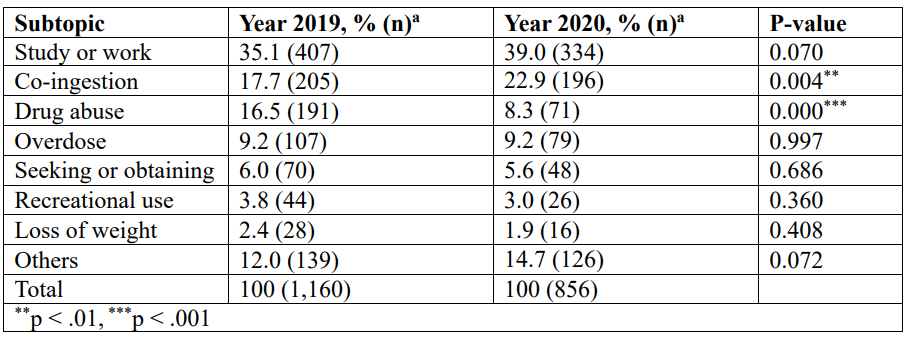


COVID-19, coronavirus disease 2019; p-value, probability value
a Data frequency of side effects and nonmedical use of methylphenidate was analyzed by allowing duplication

**Figure S1. Wordcloud to highlight the words after preprocessing the firsthand experience data
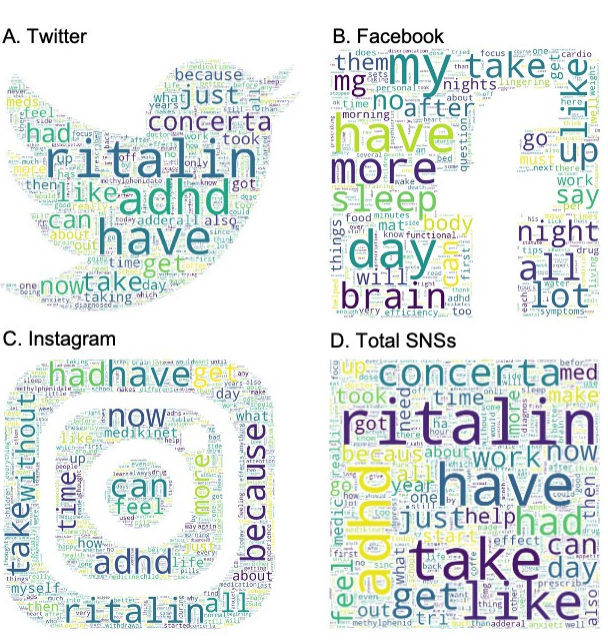
**
